# Genomic variation in *Plasmodium relictum* (lineage SGS1) and its implications for avian malaria infection outcomes: insights from experimental infections and genome-wide analysis

**DOI:** 10.1186/s12936-024-05061-3

**Published:** 2024-08-29

**Authors:** Victor Kalbskopf, Justė Aželytė, Vaidas Palinauskas, Olof Hellgren

**Affiliations:** 1 Evolutionary Ecology and Infection biology, Department of Biology, Lund University, Lund, Sweden; 2 Nature Research Centre, Akademijos 2, 08412 Vilnius, Lithuania

**Keywords:** Avian malaria, Infection experiment, *Plasmodium relictum*, RNA-seq, Genetic variation

## Abstract

**Background:**

The globally transmitted avian malaria parasite *Plasmodium relictum* (lineage SGS1) has been found to infect hundreds of different bird species with differences in infection outcomes ranging from more or less latent to potentially mortal. However, to date basic knowledge about the links between genetic differentiation and variation in infection outcome within this single malaria parasite species is lacking.

**Methods:**

In this study, two different isolates of SGS1, obtained in the wild from two different host species, were used to investigate differences in their development in the blood and virulence in the experimentally infected canaries. Simultaneously, 258 kb of the parasite genome was screened for genetic differences using parasite mRNA and compared between experimental groups.

**Results:**

The two isolates showed differences in development and caused mortality as well as effects on the blood parameters of their hosts. Although previous studies using single genes have shown very limited within lineage genetic diversity in the European population of SGS1, 226 SNPs were found across 322 genes, which separated the two experimental groups with a total of 23 SNPs that were fixed in either of the experimental groups. Moreover, genetic variation was found within each experimental group, hinting that each avian malaria infection harbours standing genetic variation that might be selected during each individual infection episode.

**Conclusion:**

These results highlight extensive genetic variation within the SGS1 population that is transferred into individual infections, thus adding to the complexity of the infection dynamics seen in these host–parasite interactions. Simultaneously, the results open up the possibility of understanding how genetic variation within the parasite populations is linked to the commonly observed differences in infection outcomes, both in experimental settings and in the wild.

**Graphical Abstract:**

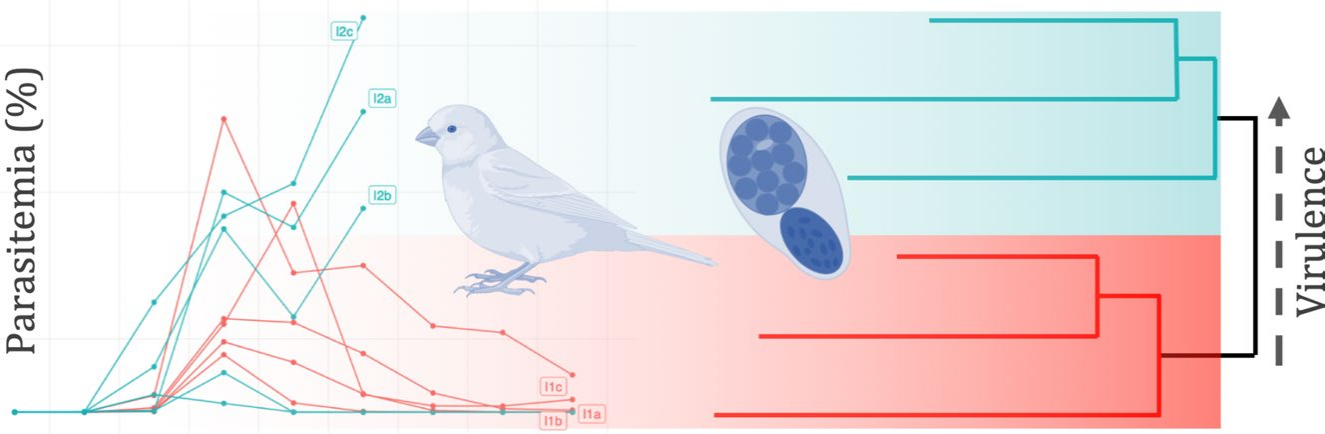

**Supplementary Information:**

The online version contains supplementary material available at 10.1186/s12936-024-05061-3.

## Background

Trying to disentangle the cause of variation in infection severity has the potential to provide fundamental insights into host–pathogen interactions. Any infection episode’s outcome can vary due to differences in the hosts, the parasites, and how the combination of variations interact. The differences in the host and the parasite can be summarized as either genetic or phenotypic variations. Previous exposure to the parasite or different physiological conditions of the host are examples of host phenotypic variation [1, 2]. To study the genetic causes of infection severity in wild animal populations, where the standing genetic variation is often substantial, large samples of the host and parasite population are usually needed to achieve statistical power [3]. Therefore, the initial step to gain insight into the complex host–parasite system can be achieved by using simplified experimental setups with closely related parasites in closely related hosts.

Avian malaria can provide such a system in which studies on parasite virulence might be achievable. Avian malaria is caused by parasites belonging to the genera *Plasmodium* and is transmitted by Culicidae mosquitoes [4, 5]. In the wild, the majority of bird species that have been investigated are infected by one or several avian *Plasmodium* parasites, which has revealed that the parasites can be found on every continent except Antarctica [5]. The host range of avian haemosporidians depends on the parasite species, which can be as broad as a few hundred host species or confined to just one species [6]. When a generalist infects a new susceptible species, it generally results in higher mortality than in an established host [7–9], though specialist avian parasites were found to be even more deadly, mainly when infecting phylogenetically distant hosts [10]. *Plasmodium relictum* is one of the most common generalists within this host–parasite system, with observed infections in 292 bird species to date [11]. Experimental studies have revealed that *P. relictum* can cause different disease severity, developing low to high intensities of parasitaemia in the blood, potentially causing acute or even deadly infections, depending on the host species [12, 13]. However, even within a species, infection outcome has a large breadth. This has further been observed when examining one of the many mitochondrial lineages that constitutes the morpho-species of *P. relictum*, i.e., the widespread mitochondrial lineage of SGS1, which has variation in infection outcomes when isolates obtained from different host individuals or species are used [14, 15]. Hellgren et al*.* [16, 17] aimed to determine the variation of SGS1 in Europe using the gene of merozoite surface protein 1 (MSP1), which is involved in the invasion of erythrocytes and is a potential target of the host’s immune system. They found no variation in this gene within the European population of the parasite, even though the MSP1 gene is often used in human malaria parasites (e.g. *Plasmodium falciparum* or *Plasmodium malariae*) as an essential marker for genetic variation and clinical outcomes [18–21].

However, there still seem to be differences in infection severity of a single genetic lineage isolated from different birds, suggesting that the isolates are not clonal. More genes should be analysed to understand the underlying host–parasite interactions at the gene level and possibly the multiclonality of an infection.

As the *Plasmodium* genome is much smaller than the host genome, and bird erythrocytes are nucleated, untargeted DNA sequencing is extremely costly and challenging. However, RNA sequencing has been used successfully in the past since the ratio of host to parasite RNA is more favorable [22]. Garcia-Longoria et al*.* [23] showed differential expression between two different host species (starling and crossbill) in *Plasmodium homocircumflexum* (lineage COLL4) infections, demonstrating a plastic response in the parasites to different host genotypes. In a previous study [24], where multiple birds were infected with SGS1 isolate of *P. relictum* that originated from a single infected host, high variation in both gene expression and parasitaemia was observed. However, the underlying genomic variation responsible for the differences in expression still needs to be investigated. In this study, an experimental setup was used with the aim to identify the variation in infection outcome between isolates and the degree of genetic variation that occurs in expressed genes between two different SGS1 isolates of the *P. relictum*.

## Methods

### Data source

To evaluate genetic variation and the impact of two different isolates of the avian malaria parasite—*Plasmodium* (*Haemamoeba*) *relictum* (lineage SGS1, GenBank accession no. JX993045) isolated from a naturally infected common crossbill (*Loxia curvirostra*) and *P. relictum* (lineage SGS1, GenBank accession no. JX993045) isolated from naturally infected house sparrow (*Passer domesticus*) on birds’ health, data from two previous studies conducted by Palinauskas et al. [15, 25] were analysed and compared. Both studies investigated the dynamic patterns of *P. relictum* SGS1 during single and co-infections with implications for host health. This study used blood samples of *P. relictum*-infected birds from the 12th day of experiments to investigate genomic differences between two *P. relictum* SGS1 isolates. The infection development and effects on host health were compared between *P. relictum*-infected groups of two experiments. Palinauskas et al. [15, 25] comprehensively describe the experimental design and procedures. The Lithuanian State Food and Veterinary Service approved the experimental procedures described herein (No. 2015/05/07-G2-27).

### Experimental design

Briefly, infections were multiplied and cryopreserved in liquid nitrogen as described by Dimitrov et al. [14]. Birds from I1 and I2 groups received *P. relictum* isolates from naturally infected common crossbill and house sparrow, respectively, which had been passed four and three times between hosts, respectively, prior to this experiment. Experimental studies were carried from September to October in 2015 and 2017, respectively, at the Nature Research Centre, Vilnius, Lithuania. The domestic canaries, *Serinus canaria domestica* were purchased commercially and maintained in a vector-free vivarium under controlled conditions (11/13 h light/dark photoperiod, 21 ± 1 °C). All birds were kept in separate cages, the food and water were provided ad libitum*.* In both experiments, canaries were randomly allocated to infected and control groups, with 6 birds in each. Infected groups I1 and I2 received a total of 7 × 10^4^ and 3 × 10^5^ RBCs with *P. relictum* meronts, respectively, while control birds received uninfected blood. Every 4 days post-infection (p.i.) the blood samples were taken to monitor birds’ health and blood parameters.

### Statistical analysis of blood and health parameters

The health parameters’ data of experiments that lasted for 32 days p.i. was compared between the groups infected with different isolates of *P. relictum* SGS1 [15, 25]. Statistical analysis was performed using R 3.6.1 [26]. A Mann–Whitney U test (a non-parametric test for comparing independent variables between two groups) was used to compare parasitaemia at the peaks of infection of I1 and I2 birds and blood parameters between the experimental groups, I1 and I2. A linear mixed model was employed to assess the statistical relationship between the response variables, namely the percentage of polychromatic red blood cells (RBCs) and haematocrit (HCT), and the predictor variables, including haemoglobin (Hb), haematocrit, parasitaemia, weight, and time. Individual birds were treated as random effects within the model. Inspection of model residuals revealed that it did not violate major model assumptions. Based on the normality and skewness of the data, some parameters’ values were transformed (e.g., square root transformation). A *P* value of 0.05 or less was considered significant.

### RNA extraction and sequencing

**Fig. 1:**
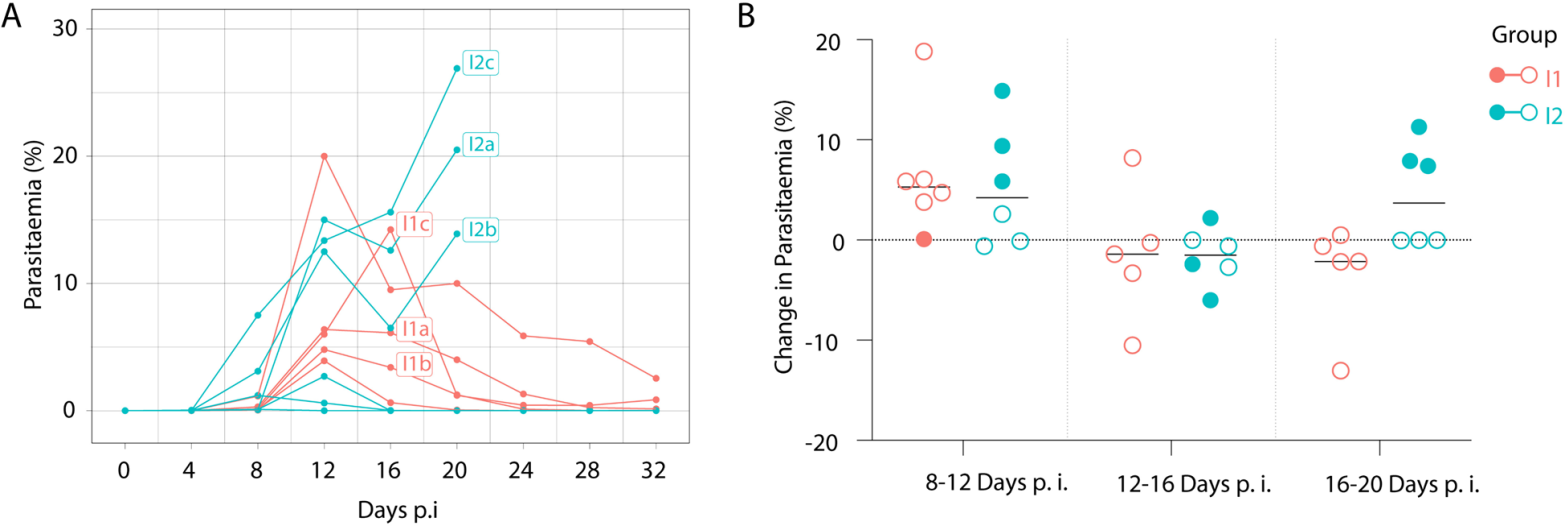
The development of parasitaemia of two *Plasmodium relictum* SGS1 isolates (I1 and I2). Individual values (**A**) are presented as % of infected erythrocytes. Three sequenced samples from isolates I1 and I2 were specifically labeled in squares as a, b, and c. Changes in parasitaemia (**B**) of survived (hollow circles) and those that died (solid circles) during the experiment are shown

Three birds from each experimental group were selected for RNA sequencing based on having high enough parasitaemia in order to obtain sufficient RNA coverage. Blood samples for RNA analysis were taken during peak parasitaemia 12 dpi for all birds (Fig. 1A). For a detailed description of the RNA extraction, library preparation, and sequencing protocol, see Garcia-Longoria et al. [23]. Briefly, total RNA was extracted from whole blood using the TRIzol (Invitrogen Carlsbad, CA) and chloroform method. The post centrifugation supernatant was treated using an RNeasy Mini Kit (Qiagen, GmbH, Hilden, Germany) following the manufacturer’s instructions. Novogene, Hong Kong, performed RNA quality control, DNAse treatment, rRNA reduction, and amplification using the SMARTer Ultra Low kit (Clontech Laboratories, Inc.) and then completed the library preparation and cDNA synthesis. Sequencing was performed on the Illumina HiSeq 2000 system. The reads were demultiplexed and screened using FastQC ver 0.10.1 [27].

### Sequence analysis

To reduce the number of host mRNA reads, the samples were filtered using the FastqPuri ver 1.0.8 Bloom filter [28] using the *Serinus canaria* genome (*Cibio_Scana_2019—Genome—Assembly—NCBI*, 2019). Adapters and low-quality reads were removed by Trimmomatic ver 0.39 [29] using the following settings: ILLUMINACLIP: TruSeq3-PE.fa:2:30:10:8:TRUE SLIDINGWINDOW:4:15. The filtered and trimmed fastq files were mapped to the reference *Plasmodium relictum* SGS1-like genome version 54 [30] using HiSat2 [31]. The resulting BAM files were sorted (by position) and indexed using SAMtools ver 1.14 [32]. BCFtools [33] was used to create consensus genomes for each file by calling (mpileup and call commands) and filtering variants in each BAM file. In parallel, a bed file was created that included regions with at least 15 reads of depth across all BAM files using SAMtools and Bedtools v2.30.0 [34] and GNU Awk ver 5.0.1. Bedtools used the bed file to filter the consensus fasta files made earlier. This resulted in one fasta file per sample that only includes regions that were supported by at least 15 reads in all samples. This step was also performed on the reference genome so the samples could be compared to the reference.

### Polymorphisms and phylogenetics

A multiple alignment was performed using MUSCLE [35] for each partial gene, and gene names were assigned by gffutils [36] using the coordinates provided by Bedtools. The multiple alignments (MA) were trimmed and filtered to remove genes without polymorphisms and partial genes with a high single nucleotide polymorphism (SNP) to length ratio (> 0.1), with the Biopython module [37]. The alignments with a high SNP to length ratio were generally short junk alignments and represented low quality data. Multiple alignments with the reference included were used to construct the maximum likelihood phylogenetic tree. ModelFinder [38, 39] was used in IQ-TREE ver 1.6.12 [40] with the ‘-m TEST’ parameter to determine the optimum genetic model for each partition. The SH-aLRT bootstrap parameter was set to 5000, and the partition file was created by Seqkit ver 2.0.0 [39]. The corresponding sequence from the reference genome was chosen as the outgroup. To identify the genes and SNPs that uniquely distinguish the isolates from each other, an UpSet plot was created using the MAs without the reference and gaps in R ver. 4.1.2 with the libraries bios2mds ver 1.2.3 [41], tidyverse ver. 1.3.1 [42], and UpSetR ver. 1.4.0 [43]. The genes with SNPs that were unique to each isolate group were selected for GO enrichment analysis. These SNPs were selected because they indicated group identity, i.e., what makes the isolate distinct when compared to the other isolate. Each SNP was manually annotated in Geneious Prime (Ver. 2019.2.3) to determine whether or not they were synonymous.

## Results

### Development of *Plasmodium relictum* blood stages and virulence in the host

All recipients in the I1 and I2 groups developed infections of different *P. relictum* SGS1 isolates obtained from crossbill and house sparrow, respectively (Fig. 1A). The mean pre-patent period during infections was, on average, 8 ± 0 days p.i. for I1 infected birds and 4 ± 0 days p.i. for I2 infected birds. On average, the peaks of parasitaemia were observed between 12 and 16 days p.i. and between 8 and 20 days p.i. in birds from I1 and I2 groups, respectively. The mean peak of parasitaemia insignificantly differed between I1 (8.2%) and I2 (10.9%) birds (W = 18, *P* = 1). However, the pattern of the development of parasitaemia was somewhat different for the two isolates (Fig. 1A). Parasites from the I1 group had the peak of parasitaemia and decreased to the chronic level, while parasites in the I2 group birds developed peaks of parasitaemia, decreased, and then went up in three individuals finally causing the death of the hosts (Fig. 1A). One bird from the I1 group died at 8 days p.i. without developing high-intensity of parasitaemia, likely unrelated to the infection (Fig. 1B), while the remaining birds survived until the end of the experiment, 32 days p.i. Half of the birds from the I2 group died on the day when parasitaemia peaked, while the other half survived as the parasitaemias were suppressed and went down to chronic level (Fig. 1B). Those birds that died in the I2 group developed the highest peaks of parasitaemia 12 days p.i. (Fig. 1A, B).

**Fig. 2:**
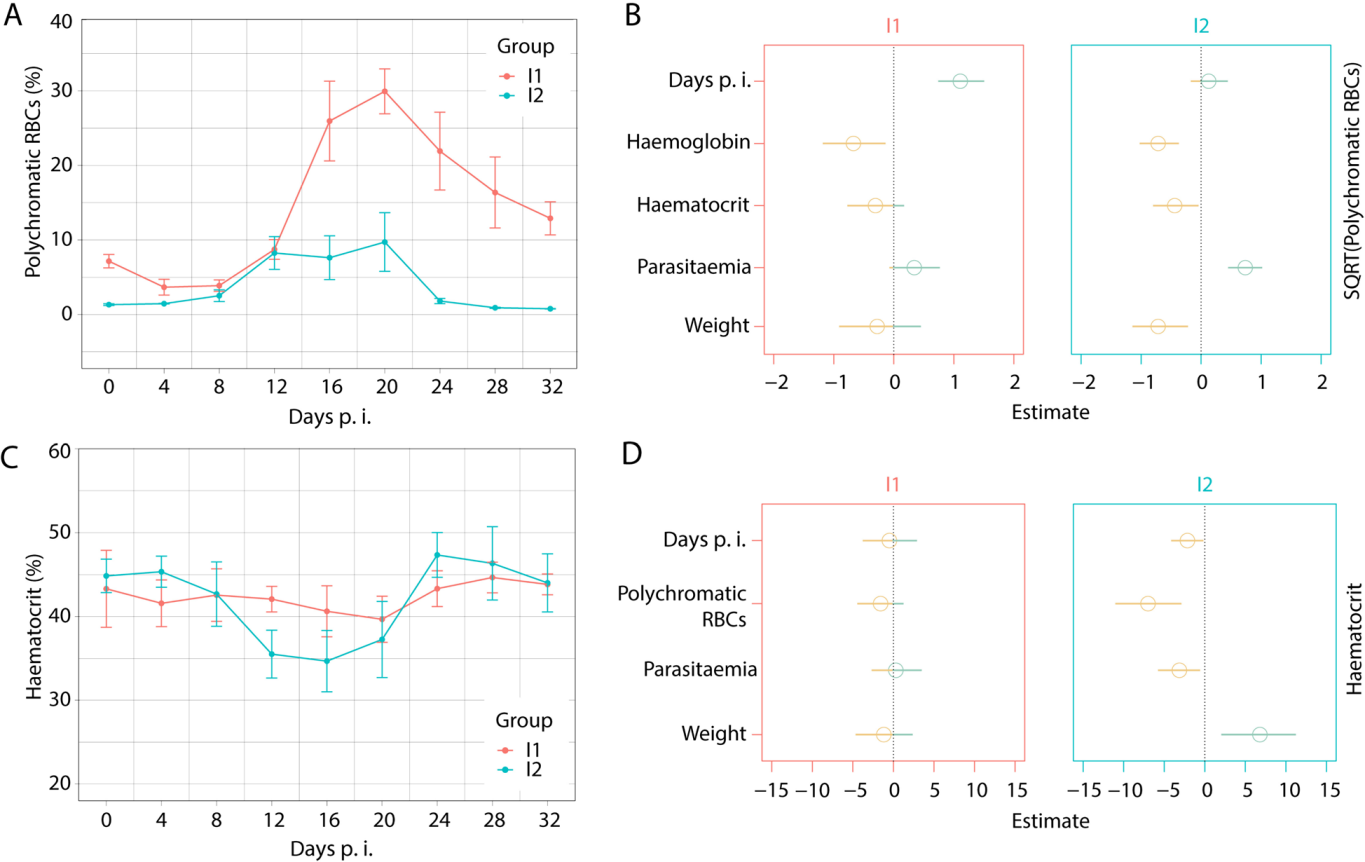
The development of polychromatic red blood cells (RBCs) (**A**) and haematocrit level (HCT) (**C**) in birds infected with two *Plasmodium relictum* SGS1 isolates (I1 and I2). Estimated mean values with standard deviation of the response variables, polychromatic RBCs (**B**) and HCT (**D**), over time showing correlation with predictor values of Haemoglobin, HCT, parasitaemia, weight or polychromatic RBCs, respectively

The increased production of polychromatic erythrocytes, which indicates the regenerative capacity of erythrocytes, was observed in birds from both I1 and I2 groups (Fig. 2A). The difference in polychromatic RBCs between the two groups was significant (W = 1686.5, *P* < 0.0001). Starting from 16 days p.i., the I1 group showed significantly higher regenerative capacity, even with slightly lower (not significantly) peaks of parasitaemia. An abnormal increase in the number of polychromatic RBCs was present in I1 birds. The values of haemoglobin negatively correlated with the number of polychromatic RBCs in both experimental groups (Fig. 2B). However, it seems that a slight increase of polychromatic RBCs in I2 birds was insufficient to recover from infection. The changes in HCT were observed in both experimental groups, with a prominent decrease in the value of HCT during the peaks of parasitaemia of the I2 group (Fig. 2C). There were no differences in Hb or HCT between the I1 and I2 groups (*W* = 1162.5, *P* = 0.3; W = 1038.5, *P* = 0.962, respectively). However, the responses of a health parameter, HCT, to various predictors, including parasitaemia, timing, polychromatic RBCs, and weight, were significant only in the I2 group (Fig. 2D). The detailed analysis of multiple predictors enabled us to untangle differences between different *P. relictum* SGS1 isolates, even when the peaks of developed parasitaemia did not differ significantly between infected groups.

### Sequence analysis

The RNA-seq resulted in a mean of 7,673,429 paired reads with a standard deviation (SD) of 2,402,901 reads (Supplementary Fig. 1, Supplementary Table 1). Mapping the reads with HiSat2 resulted in a mean of 1,590,093, uniquely mapping reads with an SD of 975,806 reads (Supplementary Fig. 2, Supplementary Table 2).

The process of creating the consensus regions with at least 15 reads of support across all samples resulted in 745 regions with enough sequencing depth to compare between individuals, with a total length of 258,513 bp that was comparable between all the samples. Of the 745 regions, 332 regions (Appendix 1) had SNPs that were considered high quality alignments (Supplementary Fig. 3) where the SNP to length ratio was below 0.1. This threshold was chosen as it delineated between variation caused by inappropriate alignments and orthologous alignments. Thirteen of the 332 regions did not map to a region with an annotated gene, lending support for the presence of unmarked genes or pseudo-genes.

### Polymorphisms and phylogenetics

**Fig. 3:**
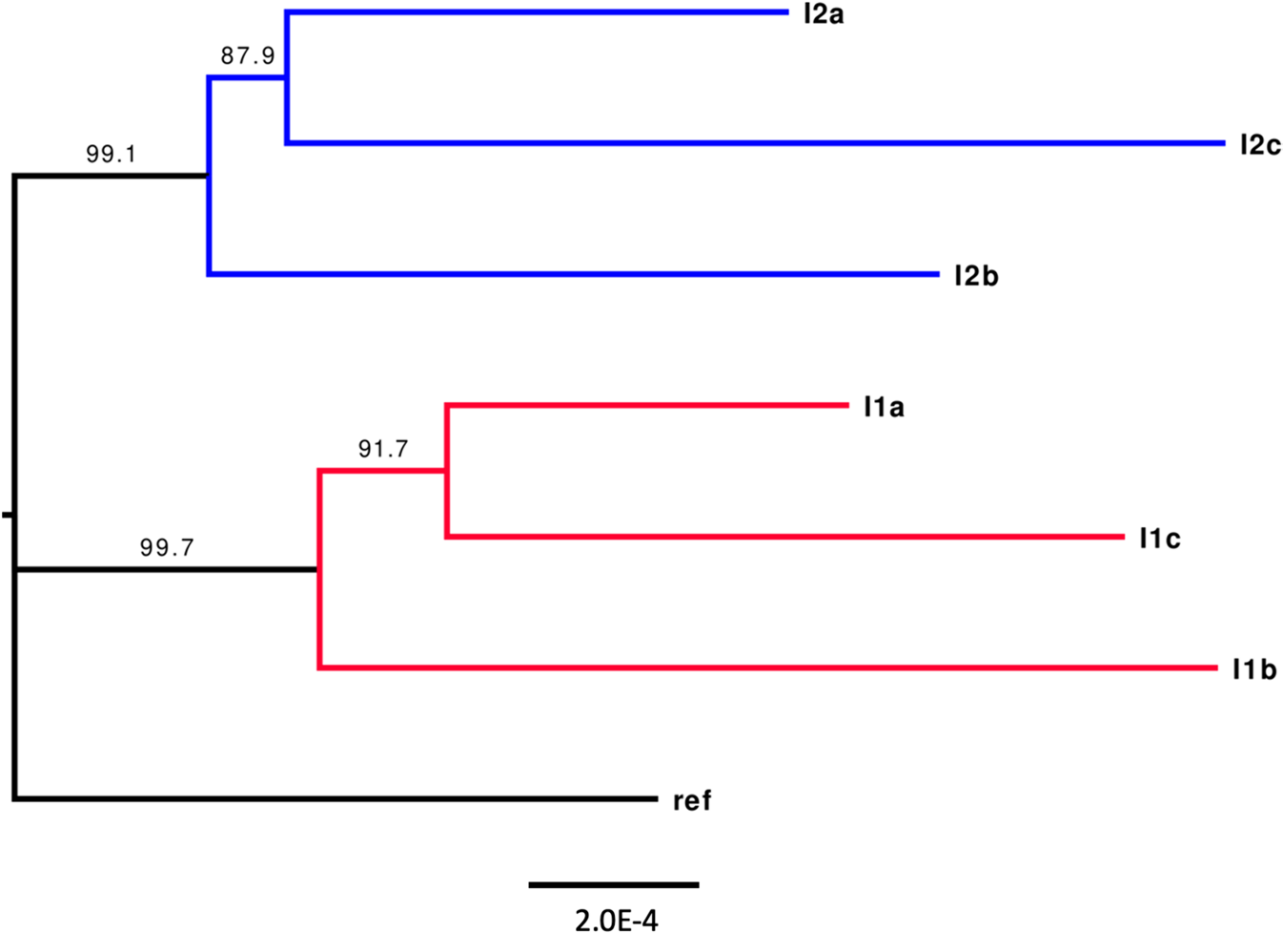
Phylogenetic tree of the multiple alignment of expressed genes of *Plasmodium relictum* in their hosts with the reference genome (*P. relictum* SGS1 Plasmodb release 54) as out group. The I1 and I2 groups are SGS1 infections isolated from two different hosts. Values on the branch represent SH-aLRT (approximate likelihood ratio test) values, and values > 95 are considered good branch support. The scale bar represents the average number of nucleotide substitutions per site

**Fig. 4:**
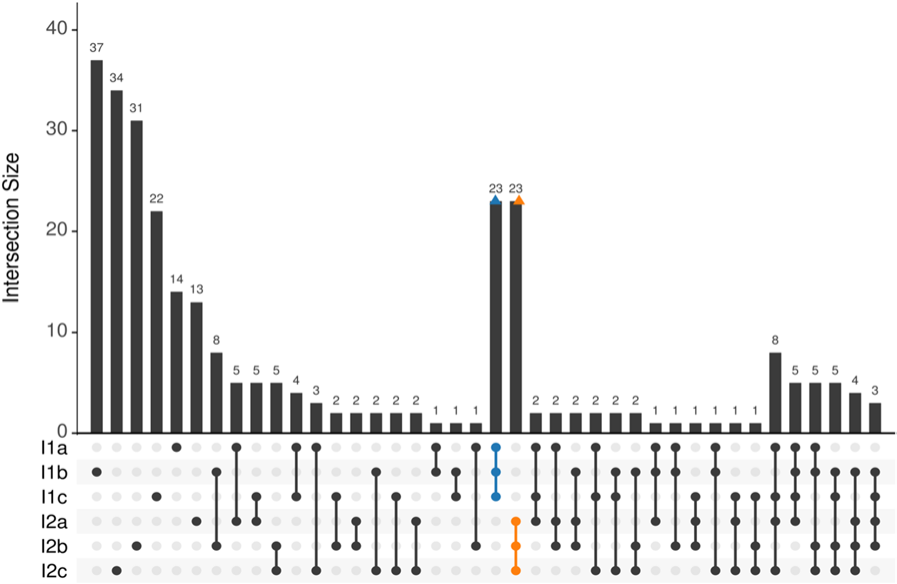
Upset plot of sets of SNPs present in each sample. Each sample has its unique set of SNPs, but the isolate groups, highlighted in blue and orange, share a distinctly larger (23) set of SNPs than any other group of samples. The plot also shows variation shared within and between groups

A total of 226 SNPs were found throughout the dataset when excluding the reference genome to which the data was mapped to, i.e. across 258,513 bp of between sample comparable data, with a total of 117 and 124 SNPs being identified within group I1 and I2, respectively. The mean number of SNP between samples within groups was 78 and 83 in I1 and I2, with a between group average of 101.2 SNPs. The tree (Fig. 3) showed strong branch support for a separation between groups I1 and I2 with SH-aLRT (Shimodaira–Hasegawa approximate likelihood ratio test) support values of 99.7% and 99.1% respectively. An UpSet plot (Fig. 4) shows the distribution of the SNPs identity between all the samples and clearly indicates a peak in the SNPs distribution with 23 SNPs that are exclusive and fixed between the I1 and I2 groups. The next largest group of exclusive SNPs shared between at least 2 samples has only 8 SNPs. The 23 group exclusive SNPs were present in 20 genes, 7 of which are synonymous and 13 non-synonymous (Supplementary Table 3). The concatenated partitioned alignments were used to produce a phylogenetic tree (Fig. 3). ModelFinder indicated that the F81 + F (base frequencies are allowed to vary) is the best model for 317 of the 332 partitions using the Bayesian information criterion (BIC) scores and weights.

Although the GO term enrichment analysis showed significant results (Supplementary Table 4), the terms involved did not reveal an obvious causative pathway for the observed differences between the infection groups.

## Discussion

Although SGS1 is one of the most widespread malaria lineages of *P. relictum*, it has been thought to exhibit a monomorphic population structure in Europe, based on data from its merozoite surface protein 1 (MSP-1, an autosomal gene) [17]. This has led to speculations that SGS1 has undergone a pandemic population expansion in Europe, resulting in low or very little genetic variation within its population. However, previous studies have shown that SGS1 isolates obtained from different donor birds from the same geographical region might show differences in infection properties [12, 44]. Using a unique infection approach that involved monitoring the blood properties of infected birds and analysing the transcriptome of the parasites, it was demonstrated that SGS1 isolates from different donors (I1 and I2) exhibit distinct infection properties when infecting new individuals. Additionally, fixed genomic differences were observed in the parasites between birds infected with each isolate. Moreover, within each infection group, the parasites also showed genetic variation between the recipient birds, even though the infection originated from a single donor. However, less variation was found within infection groups. Some of this variation was also shared across the infection groups, suggesting that standing genetic variation occurs in the wild parasite and is represented within single infection episodes. In this study, this variation had not become fixed within each of the isolates used for the two different infection groups. This further suggests that each infection is a transfer of a “population of haplotypes” that contains standing genetic variation and not just a transfer of identical haploid clones (Fig. 4) and that different variants might become the most dominant, although the source infection is the same.

### Development patterns and host response to the experimental infections with different *Plasmodium relictum* isolates

*Plasmodium relictum* SGS1 isolates obtained from crossbills and house sparrows developed infections in experimentally infected canaries in a similar manner at the beginning, reaching the initial peaks of parasitaemia with broad individual variation (Fig. 1A). This resulted in insignificant differences in average peak parasitaemia between the two SGS1 isolates. However, after reaching the peak of parasitaemia, the development patterns diverged for the two isolates (Fig. 1A). Parasites from the I1 group reached a peak of parasitaemia and then decreased to chronic levels. Meanwhile, parasites in the I2 group birds developed peaks of parasitaemia, declined, and then, in some cases, parasitaemia surged uncontrollably, peaking again and causing the death of the host (Fig. 1A). The development pattern of *P. relictum* SGS1 isolates in other susceptible bird species is similar after experimental infection, typically causing varying peaks of parasitaemia before stabilizing at a chronic level [12, 44–49]. However, individual variations in the development patterns are often observed within groups of birds infected with the same number of parasites obtained from the same donor [44, 46]. These variations may reflect differences in the physiological and immune statuses of each recipient, or they could also result from a combination of host health background and genetic diversity of *P. relictum* clones within the inoculated dose [44].

The survival of experimentally infected birds in both groups, I1 and I2, was similar on average—28.5 and 26 days p.i., respectively. However, in the I1 group, only one bird died at the beginning of the experiment without developing high parasitaemia. In contrast, in the I2 group, half of the birds (three birds) died when parasitaemia peaked later during the experiment (Fig. 1B). A direct comparison of survival between the two groups does not always reveal the nuances of infection differences, necessitating a more detailed interpretation of the results (Fig. 1B). Those birds that died in the I2 group developed the highest peaks of parasitaemia at 12 days p.i. (Fig. 1A, B), and after a temporary suppression, parasites might have circumvented the immune system and multiplied to uncontrolled lethal levels. Such cases, where parasites proliferate and cause extremely high levels of parasitaemia leading to the death of the hosts, were also recorded in other experiments with siskins infected with different isolates of the SGS1 [44, 46].

Haemoglobin and haematocrit levels decreased in both I1 and I2 birds, consistent with observations in other studies where siskins were infected with different SGS1 isolates [12, 44], highlighting the sensitivity of these haematological measures to *Plasmodium* infections. A detailed analysis of various predictors helped elucidate differences between the *P. relictum* SGS1 isolates, revealing significant responses of HCT to predictors such as parasitaemia, timing, polychromatic RBCs, and weight, primarily in the I2 group (Fig. 2D). Notably, the correlation between HCT values and parasitaemia, as well as polychromatic RBC levels, was absent in the I1 group, possibly due to the slight HCT decrease observed as the number of polychromatic RBCs and infected erythrocytes increased (Figs. 1A, 2A, C), while the decline in HCT occurred alongside increases in parasitaemia and polychromatic RBCs levels during 12–20 days p.i. in the I2 group.

The number of polychromatic RBCs, or immature RBCs, in the blood, together with haematocrit values, can be used to gauge regenerative capacity [50]. The proportion of polychromatic RBCs in healthy birds varies depending on the species but typically does not exceed 10% of the total RBC count. An increased number of polychromatic RBCs, a condition known as polychromatophilia, is common in vertebrates during recovery from anaemia, including recoveries following peaks of *Plasmodium* infections [4, 51]. It is also an important indicator of bone marrow activity. The percentage of polychromatic RBCs within the first 8 days p.i. was within normal limits in both groups, with a slight increase towards 12 days p.i. However, after day 12, the rate of polychromatic RBCs diverged between the two groups (Fig. 2A). In group I1, birds exhibited increased polychromatic RBCs until the experiment’s end, with a slight decrease towards 32 days p.i., when parasitaemia subsided to chronic levels. Conversely, in group I2, where an extreme increase in parasitaemia was recorded in three infected birds, the number of polychromatic RBCs did not increase compared to day 12. The insufficient regenerative capacity in group I2 may have contributed to the death of infected birds. It remains unclear what caused these differences in regenerative capacity and erythrocyte production between I1 and I2 birds and why increased parasitaemias and RBC destruction in I2 birds did not lead to higher cell regeneration. It can be speculated that malarial parasites disrupted the erythropoiesis process. It is known that some avian malarial parasites develop in the bone marrow and can interrupt the erythropoiesis process [15]. Parasites such as *Plasmodium elongatum* and others in the subgenus *Huffia* develop in immature erythrocytes and haematopoietic tissues [4, 5] and may disrupt erythrocyte production. A recent study observed a similar impact on erythropoiesis, where extreme non-regenerative anaemia occurred during the infection of canaries with the *P. elongatum* ERIRUB01 parasite, leading to the death of 5 out of 6 infected birds [15]. It was also shown that this parasite heavily infects bone marrow cells and can occasionally be found in other haematopoietic organs, such as the liver and spleen [52]. However, *P. relictum*, belonging not to *Huffia* but the *Haemamoeba* subgenus, does not exhibit secondary exoerythrocytic (phanerozoites) development in bone marrow [5]. Only primary exoerythrocytic merogony in bone marrow is described for *P. relictum*, which occurs after the host is infected with sporozoites [5]. Since our infection experiments are based on inoculation of infected blood, none of the primary exoerythrocytic stages (cryptozoites and metacryptozoites) should be detected. Although secondary exoerythrocytic stages of *P. relictum* have never been recorded in bone marrow previously by using traditional methods, our results indicating the effect on the haematopoiesis call for more detailed analyses. The most recent study employing in situ hybridization to identify *P. relictum* SGS1 parasite stages in organs successfully detected only blood stages in bone marrow and other organs. However, it did not clarify whether SGS1 parasite undergoes secondary exoerythrocytic merogony [53]. Advancements in in situ hybridization methodologies and the application of immunofluorescent diagnostics might be helpful for more precise studies. Recent studies in human malaria have highlighted the critical role of haematopoietic organs, particularly the bone marrow, as a niche for gametocyte development and a reservoir for asexual *Plasmodium* stages, underscoring the underestimated importance of these organs [54]. It is possible that not only avian *Plasmodium* belonging to subgenus *Huffia*, but also other avian malaria parasites may influence erythropoiesis through mechanisms that have yet to be fully elucidated.

### Genetic differences between the parasites of the infected hosts

When observing differences in infection outcomes, as in this case, either in terms of mortality or differences in blood parameters, the question arises whether the degree to which this can be linked to genetic differences of the parasite or not. In the case of *P. relictum* (SGS1), it is considered to consist of a single recombining population in Europe [17]. The degree to which multiple genetic lineages within the population might affect the host differently is expected but hasn’t been investigated to date. In this study, discreet genetic variation was observed between the two infection groups, and genetic variation not explained by the isolates. Twenty-three SNPs in 20 genes were fixed between the two infection groups. The variation that is exclusive between the two infection groups might be due to species specific selection of certain haplotypes prior to the infection experiment based on the immune systems of the donor birds in the wild (i.e., the crossbill and house sparrow) from which the parasites where obtained. Alternatively, the fixation of haplotypes between the infection groups might solely be an effect of a random bottleneck event that happened in nature when the birds were originally infected by a mosquito or artificially during the inoculation of the donor birds in an analogous way that can occur when infected by a vector (i.e. when inoculating the experimental birds only a subset of all parasites might be injected and only a subset of those might be responsible for starting the infection). Experimental infections by direct inoculation, as used in this study, may reveal greater variation compared to what is found in wildlife infections as the parasite haplotypes of which the infection comprise are not subject to selection events in the mosquito, which in turn is known to create a significant bottleneck effect [55]. However, the recombination event that occurs in the mosquito might counterbalance these bottlenecks by creating new sets of genotypes within the infection population.

Although variation at 23 sites that had gone to fixation between the isolates was identified, no conclusions could be drawn as to whether those specific genes themselves are responsible for the observed differences in infection effects between the infection groups. As the genetic variation observed in this study is a representation based on 258 kbp, which only accounts for 1% of the whole parasite genome, the observed genetic differences are likely a reflection of linked genetic variation found throughout the genome of the different haplotypes. However, the results obtained here highlight the need for future studies using methods that allow for the sequencing of larger proportions of the parasite genomes. This would allow us to find segregated genomic regions linked to different infection outcomes, thus increasing our understanding of the genetic links to the variation in infection severity observed in the wild and during experimental setups [45].

Moreover, although some variation is fixed between infections, there is also variation that has not gone to fixation neither within infection groups where the infection originates from the same donor bird nor between individuals that received their infection from different donor birds. Although part of the genome has become fixed between the isolates, there still exists much variation left between individual parasites within infection. The variation that is shared across the different isolates might be due to a common evolutionary past or because they still are part of a recombining population, but when being transferred into different host species, as in this case the original donor birds, common crossbill and house sparrow, selection drives some parts of the genome to fixation. Once transferred into the recipient birds, different variants might become dominant in the new infection either through random events or selection. Important to note is that all parasites within the vertebrate host are haploid; therefore, any non-silent mutations have the potential of being selected upon as they would act as dominant alleles (excluding epistatic effects). This increases the phenotypic effect of any genetic variation when comparing samples, potentially creating a strong basis for selection. In a larger study including more infected individuals and sequencing larger parts of the parasites’ genomes, the ultimate goal would be to link specific dominant variants with infection outcomes [56, 57]. As this study has shown, the variation is there; the next step is to pinpoint it to specific genes.

### Supplementary Information

Supplementary Material 1.

Supplementary Material 2.

Supplementary Material 3.

## Data Availability

Sequence data is available under accession number PRJEB67939 at ENA (European Nucleotide Archive). The blood smears obtained from experimental birds supporting the findings of this study are available upon the request from corresponding author [VP].
