# Supplementary material for: Genomic variation in Plasmodium relictum (lineage SGS1) and its implications for avian malaria infection outcomes: insights from experimental infections and genome-wide analysis
Source: Malar J. 2024 Aug 29;23:260. doi: 10.1186/s12936-024-05061-3 (PMC11360878; doi:10.1186/s12936-024-05061-3)
Supplement: Supplementary file 2 — Supplementary Material 2. [file 12936_2024_5061_MOESM2_ESM.docx]

**Supplementary Material (Supp. Fig 1-3)**

Genomic variation in *Plasmodium relictum* (lineage SGS1) and its implications for avian malaria infection outcomes: insights from experimental infections and genome-wide analysis

## Kalbskopf V, Aželytė J2, Palinauskas* and Hellgren O*


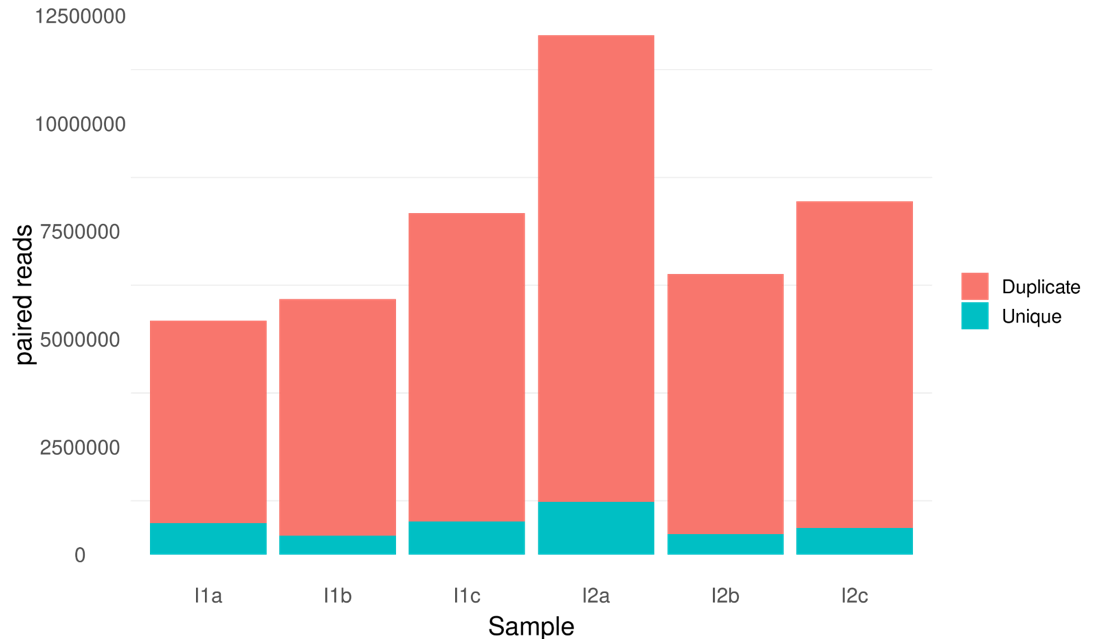


**Supplementary Figure 1.** Graph of parasite reads present in each sample after filtering, representing table 1.


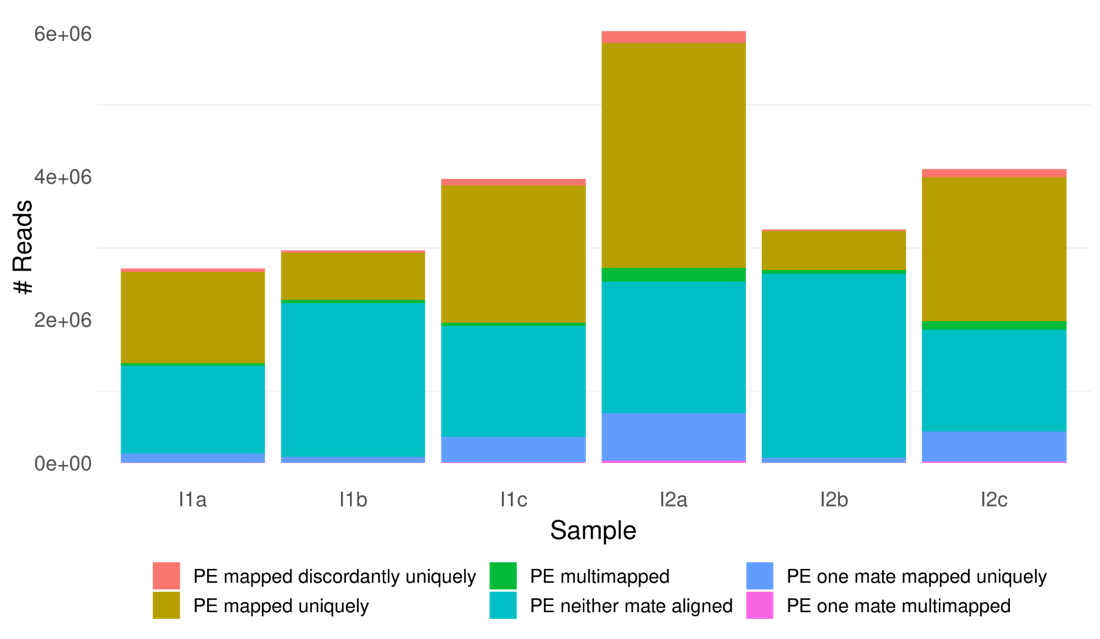


**Supplementary Figure 2.** Graph of mapping statistics for each sample. Discordant mappings occur when one read in a pair maps further away than the expected insert size, especially when they map to different chromosomes. Multimapping is caused by a lack of unique mapping coordinates.


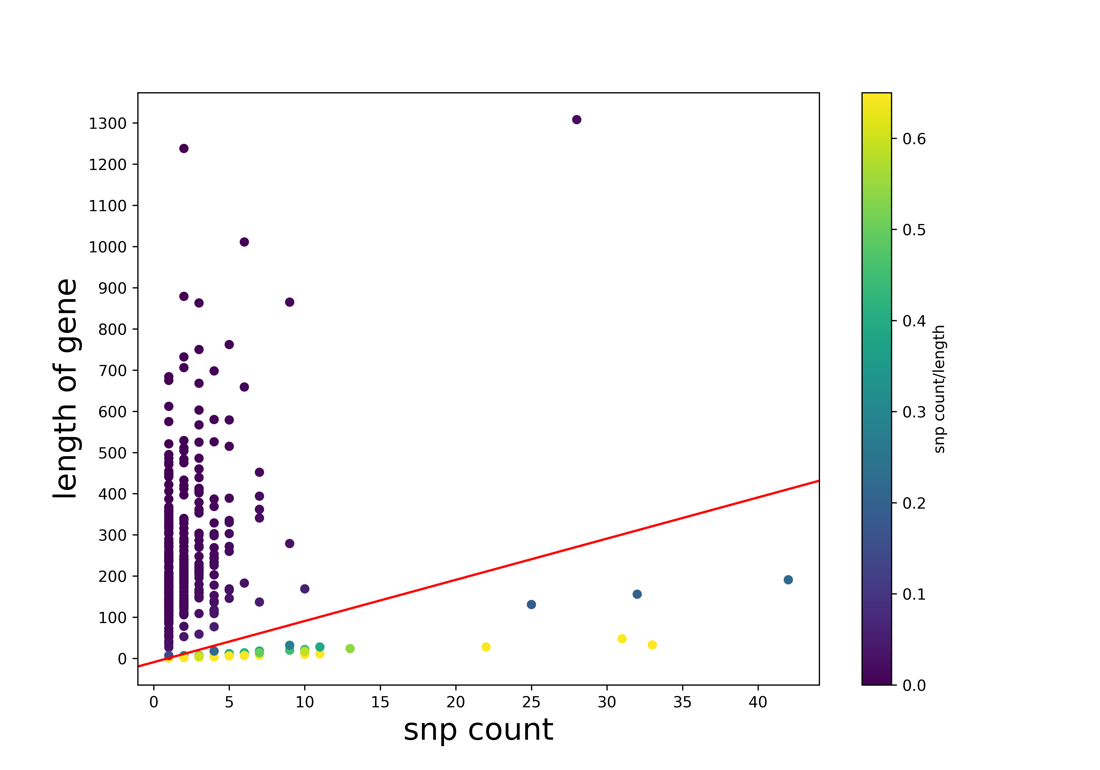


**Supplementary Figure 3.** Plot of the length of each alignment vs the number of SNPs in that alignment. Alignments with a ratio of 0.1 SNPs per nucleotide or greater (below the red line) were removed from further analysis as they were considered to be uninformative.
