# Supplementary material for: Genomic variation in Plasmodium relictum (lineage SGS1) and its implications for avian malaria infection outcomes: insights from experimental infections and genome-wide analysis
Source: Malar J. 2024 Aug 29;23:260. doi: 10.1186/s12936-024-05061-3 (PMC11360878; doi:10.1186/s12936-024-05061-3)
Supplement: Supplementary file 3 — Supplementary Material 3. [file 12936_2024_5061_MOESM3_ESM.docx]

**Supplementary Material (Supp. Table 1-4)**

Genomic variation in *Plasmodium relictum* (lineage SGS1) and its implications for avian malaria infection outcomes: insights from experimental infections and genome-wide analysis

## Kalbskopf V, Aželytė J2, Palinauskas* and Hellgren O*

**Supplementary Table 1.** The number of reads present in each sample after filtering.

| Sample | Unique | Duplicate | Total |
| --- | --- | --- | --- |
| I1a | 737108 | 4690530 | 5427638 |
| I1b | 434991 | 5497347 | 5932338 |
| I1c | 771408 | 7153132 | 7924540 |
| I2a | 1222108 | 10822366 | 12044474 |
| I2b | 476813 | 6035233 | 6512046 |
| I2c | 619657 | 7579881 | 8199538 |

**Supplementary Table 2.** Mapping statistics for each sample. Discordant mappings occur when one read in a pair maps more than the expected insert size, especially when they map to different chromosomes. Multimapping is caused by a lack of unique mapping coordinates.

| Sample | PE mapped uniquely | PE mapped discordantly uniquely | PE one mate mapped uniquely | PE multimapped | PE one mate multimapped | PE neither mate aligned |
| --- | --- | --- | --- | --- | --- | --- |
| I1a | 1273750 | 51872 | 127563 | 35775 | 3232.5 | 1224818.5 |
| I1b | 656893 | 34607 | 76316.5 | 49242 | 5482 | 2147284.5 |
| I1c | 1916579 | 92266 | 350089.5 | 49699 | 10549 | 1548038.5 |
| I2a | 3139150 | 168907 | 659840.5 | 186774 | 32330 | 1843293.5 |
| I2b | 539550 | 26922 | 67091.5 | 51008 | 4168.5 | 2571125 |
| I2c | 2014636 | 112304 | 419489 | 118948 | 19376.5 | 1419438.5 |

**Supplementary Table 3.** Annotations for the SNPs that were fixed in either of the infection groups.

| **Gene ID** | **Product Description** | **Mutation** | **AA change** | **AA position** |
| --- | --- | --- | --- | --- |
| **PRELSG_0418800** | protein kinase, putative | non-synonymous | ASP, GLU | 3rd position |
| **PRELSG_0803800** | IMP1-like protein, putative | synonymous |  | 3rd position |
| **PRELSG_0915900** | tRNA m(1)G methyltransferase, putative | synonymous |  | 3rd position |
| **PRELSG_1132700** | ribonuclease, putative | synonymous |  | 1st position |
| **PRELSG_0103400** | inositol-phosphate phosphatase, putative | synonymous |  | 3rd position |
| **PRELSG_0923800** | alternative splicing factor SR-MG, putative | synonymous |  | 3rd position |
| **PRELSG_1314900** | basal complex protein BCP1, putative | non-synonymous | Glu, Asp | 3rd position |
| **PRELSG_1144900** | trophozoite exported protein 1, putative | not determined |  |  |
| **PRELSG_0020800** | 28S ribosomal RNA | non-synonymous | Arg, Lys | 2nd position |
| **PRELSG_1207400** | DnaJ protein, putative | non-synonymous | Asn, Asp | 1st position |
| **PRELSG_1228400** | conserved Plasmodium protein, unknown function | non-synonymous | Glu, Lys | 1st position |
| **PRELSG_1342000** | aldo-keto reductase, putative | synonymous |  | 3rd position |
| **PRELSG_0208700** | conserved Plasmodium protein, unknown function | non-synonymous | Met, Ile. | 3rd position |
| **PRELSG_1228600** | conserved Plasmodium protein, unknown function | non-synonymous | Lys, Arg | 2nd position |
| **PRELSG_1311500** | coiled-coil domain-containing protein 124, putative | non-synonymous | Lys, Glu | 1st position |
| **PRELSG_1243200** | DNA repair metallo-beta-lactamase protein, putative | not determined |  |  |
| **PRELSG_0108300** | conserved Plasmodium protein, unknown function | not determined |  |  |
| **PRELSG_0110300** | serine protease DegP, putative | not determined |  |  |
| **PRELSG_0021500** | conserved Plasmodium protein, unknown function | non-synonymous | Tyr, Asp | 1st position |
| **PRELSG_1336600** | atypical protein kinase, ABC-1 family, putative | non-synonymous | Tyr, Asp | 1st position |

**Supplementary Table 4.** GO enrichment results of the genes that had SNPs which exclusively define the inoculation groups.

| **ID** | **Name** | **Bgd count** | **Result count** | **Result gene list** | **Pct of bgd** | **Fold enrichment** | **Odds ratio** | **P-value** | **Benjamini** | **Bonferroni** |
| --- | --- | --- | --- | --- | --- | --- | --- | --- | --- | --- |
| GO:0052832 | inositol monophosphate 3-phosphatase activity | 1 | 1 | PRELSG_0103400 | 100 | 287,69 | inf | 0,00348 | 0,03128 | 0,18770 |
| GO:0052905 | tRNA (guanine(9)-N(1))-methyltransferase activity | 1 | 1 | PRELSG_0915900 | 100 | 287,69 | inf | 0,00348 | 0,03128 | 0,18770 |
| GO:0052834 | inositol monophosphate phosphatase activity | 1 | 1 | PRELSG_0103400 | 100 | 287,69 | inf | 0,00348 | 0,03128 | 0,18770 |
| GO:0052745 | inositol phosphate phosphatase activity | 1 | 1 | PRELSG_0103400 | 100 | 287,69 | inf | 0,00348 | 0,03128 | 0,18770 |
| GO:0008934 | inositol monophosphate 1-phosphatase activity | 1 | 1 | PRELSG_0103400 | 100 | 287,69 | inf | 0,00348 | 0,03128 | 0,18770 |
| GO:0052833 | inositol monophosphate 4-phosphatase activity | 1 | 1 | PRELSG_0103400 | 100 | 287,69 | inf | 0,00348 | 0,03128 | 0,18770 |
| GO:0004033 | aldo-keto reductase (NADP) activity | 2 | 1 | PRELSG_1342000 | 50 | 143,85 | 310,5 | 0,00694 | 0,03748 | 0,37480 |
| GO:0009019 | tRNA (guanine-N1-)-methyltransferase activity | 2 | 1 | PRELSG_0915900 | 50 | 143,85 | 310,5 | 0,00694 | 0,03748 | 0,37480 |
| GO:0008106 | alcohol dehydrogenase (NADP+) activity | 2 | 1 | PRELSG_1342000 | 50 | 143,85 | 310,5 | 0,00694 | 0,03748 | 0,37480 |
| GO:0004032 | alditol:NADP+ 1-oxidoreductase activity | 2 | 1 | PRELSG_1342000 | 50 | 143,85 | 310,5 | 0,00694 | 0,03748 | 0,37480 |
| GO:0016423 | tRNA (guanine) methyltransferase activity | 6 | 1 | PRELSG_0915900 | 16,7 | 47,95 | 62,03 | 0,02069 | 0,10156 | 1,00000 |
| GO:0060090 | molecular adaptor activity | 8 | 1 | PRELSG_0108300 | 12,5 | 35,96 | 44,29 | 0,02750 | 0,12374 | 1,00000 |
| GO:0008175 | tRNA methyltransferase activity | 11 | 1 | PRELSG_0915900 | 9,1 | 26,15 | 30,98 | 0,03763 | 0,15630 | 1,00000 |
